# Quantum Hamiltonian algorithms for maximum independent sets

**DOI:** 10.1093/nsr/nwaf304

**Published:** 2025-07-29

**Authors:** Xianjue Zhao, Peiyun Ge, Hongye Yu, Li You, Frank Wilczek, Biao Wu

**Affiliations:** International Center for Quantum Materials, School of Physics, Peking University, Beijing 100871, China; State Key Laboratory of Low Dimensional Quantum Physics, Department of Physics, Tsinghua University, Beijing 100084, China; Department of Physics and Astronomy, Stony Brook University, Stony Brook, NY 11794, USA; State Key Laboratory of Low Dimensional Quantum Physics, Department of Physics, Tsinghua University, Beijing 100084, China; Frontier Science Center for Quantum Information, Department of Physics, Tsinghua University, Beijing 100084, China; Beijing Academy of Quantum Information Sciences, Beijing 100193, China; Hefei National Laboratory, Hefei 230088, China; Center for Theoretical Physics, MIT, Cambridge, MA 02139, USA; T. D. Lee Institute and Wilczek Quantum Center, School of Physics & Astronomy, Shanghai Jiao Tong University, Shanghai 200240, China; Department of Physics, Stockholm University, Stockholm SE-106 91, Sweden; Department of Physics, Arizona State University, Tempe, AZ 25287, USA; International Center for Quantum Materials, School of Physics, Peking University, Beijing 100871, China; Wilczek Quantum Center, Shanghai Institute for Advanced Studies, Shanghai 201315, China; Hefei National Laboratory, Hefei 230088, China

**Keywords:** maximum independent set, quantum algorithm, Rydberg atom array, non-Abelian mixing

## Abstract

We compare two quantum Hamiltonian algorithms that address the maximum independent set problem: one based on the emergent non-Abelian gauge matrix in adiabatic evolution of an energetically isolated manifold of states; the other based on designed application of single-qubit operations. We demonstrate that they are mathematically equivalent in the sense that one is the other’s interaction picture. Despite their mathematical equivalence, our numerical simulations show significant differences between them in performance, which is explained analytically. Intriguingly, this equivalence unveils that the PXP model, recently prominent in quantum dynamics research, can be viewed as quantum diffusion over the median graph of all independent sets governed by the non-Abelian gauge matrix.

## Teaser

An adiabatic quantum algorithm is shown capable of significantly outperforming earlier heuristic approach for solving the maximum independent set problem using 2D Rydberg atom array.

## INTRODUCTION

**Figure 1.**
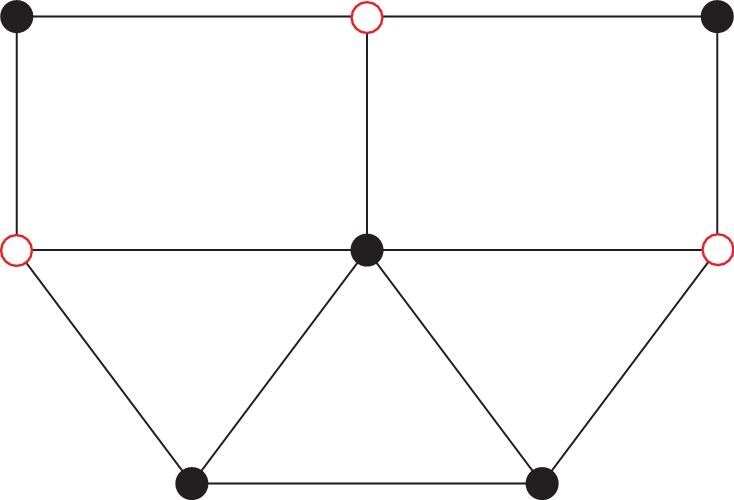
A graph with eight vertices and 12 edges. The circles stand for vertices and the lines stand for edges. The open circles form one of its maximum independent sets.

An independent set (IS) of a graph is a collection of vertices, none of which are directly connected by edges. Among all the independent sets, that with the largest number of vertices is called the maximum independent set (MIS). The red circles in Fig. 1 illustrate an MIS for a graph with eight vertices and 12 edges. Finding an MIS is an NP-hard problem on a classical computer [1]. Because of the broad prospective applications enabled by MISs, from logistics and supply chain optimization [2–4] to possible mapping into other NP-hard problems [5,6], interests in efficient and effective MIS solutions are high, especially since the first reported experimental MIS solution in a Rydberg atom quantum simulator [7]. The best-known classical algorithm has a time complexity of $1.1996^{n}n^{O(1)}$ with *n* the number of vertices in a given graph [8].

Recently, two quantum algorithms have been proposed for MISs [7,9,10]; they look quite different, although both of them employ an Ising-type interaction in their Hamiltonians. While it remains unclear whether they might offer any quantum advantage over classical algorithms, the promising scalability of the experimental platform employed in [7,11,12] raises significant hope that it may provide a viable experimental approach for finding MISs of $n>2000$.

One quantum algorithm, which we call the Peking (PK) algorithm, was introduced and refined in [9,10]. It leverages a general approach that utilizes emergent non-Abelian gauge potentials arising during adiabatic state evolution to achieve the desired interactions. The other [7], which we call the Harvard (HV) algorithm, is based on carefully designed application of one-body operators. Interestingly, we demonstrate in this work that these seemingly distinct approaches are mathematically equivalent. Specifically, the Hamiltonian used in the PK algorithm can be understood as the HV Hamiltonian transformed into the interaction picture.

Despite their mathematical equivalence, these two algorithms offer distinct perspectives that yield valuable insights. When implemented straightforwardly on Rydberg atom arrays without optimization, our numerical simulations suggest that the PK algorithm’s adiabatic path performs significantly better and requires fewer measurements in experiment. In numerical simulations for seven-vertex graphs, we find that the PK adiabatic path outperforms the brute-force segment-wise numerically optimized path in the HV algorithm by 25% and saves about $6\times 10^6$ measurements for each graph on average. Such an advantage in efficiency is found to persist for various graph types and for different sizes, raising the heightened desire for future experiments with larger sized tweezer arrays [13–22].

The equivalence also reveals a fascinating connection. The PXP model [23–29], known for its quantum many-body scarring phenomenon and experimentally realized with Rydberg atom arrays [30], is essentially the non-Abelian gauge matrix in the PK algorithm. This relationship holds significant promise. From one perspective, the PXP model, traditionally viewed as a many-body problem, can now be seen as single-particle quantum diffusion over a median graph [31,32], whose vertices represent the independent sets of the original graph. In another perspective, the PXP model, originally defined on a special one-dimensional graph, can be extended to other graphs. Such an extension could significantly enrich the exploration of quantum many-body scarring and other quantum dynamical phenomena.

## THE TWO QUANTUM ALGORITHMS

The HV algorithm [7] is referenced to the Hamiltonian


(1) \begin{eqnarray*}
\widehat{H}_{\text{HV}}&=&\frac{\hbar }{2}\sum _{j=1}^{n}\big[\Omega (t)e^{i\varphi (t)} |0\rangle _j\langle 1|_{j} \\
&&+\, {\rm H.c.} -2\Delta (t)\hat{n}_j \big] +\sum _{i<j}V_{ij}\hat{n}_i\hat{n}_j,
\end{eqnarray*}

where $|0\rangle _j$ represents that the atom at site *j* is in the ground state, while $|1\rangle _j$ represents that it is excited, and $\hat{n}_j = |1\rangle _j \langle 1|_j$. The term $V_{ij}>0$ describes the repulsive interaction between two excited Rydberg atoms at sites *i* and *j*; it imposes an energy penalty on multi-atom configurations in which both atoms at sites *i* and $j\not= i$ are excited. Each atom represents a vertex in a graph, and $V_{ij}>0$ corresponds to the existence of a line connecting vertices *i* and *j*. Here $\Omega (t)$, $\varphi (t)$ and $\Delta (t)$ are control functions of a coherent laser beam whose implementations define the algorithm. In general, the goal of such MIS algorithms is to evolve into a configuration with many disconnected excited states. These correspond to low-energy states at late times, when $\Omega \rightarrow 0$ and $\Delta$ approaches a positive constant.

With the pseudo-spin operators $\sigma _j^z=2\hat{n}_j-1$, $\sigma _j^+=|1\rangle _j\langle 0|_j$ and $\sigma _j^-=|0\rangle _j\langle 1|_j$, we can rewrite Hamiltonian $\widehat{H}_{\text{HV}}$ (up to an irrelevant time-dependent c number) as


(2) \begin{eqnarray*}
\widehat{H}_{\text{HV}}=\widehat{H}_1(t)+\widehat{H}_2,
\end{eqnarray*}

where


(3) \begin{eqnarray*}
\widehat{H}_1(t)&=& \frac{\hbar }{2}\Omega (t)\cos {\varphi (t)}\sum _j \sigma _j^x
+\,\frac{\hbar }{2}\Omega (t) \\
&&\times \, \sin {\varphi (t)} \sum _j \sigma _j^y -\frac{\hbar }{2}\Delta (t)\sum _j \sigma _j^z\\
\end{eqnarray*}

and $\widehat{H}_2=\sum _{\langle i,j\rangle }V_{ij}\hat{n}_i\hat{n}_j$. Here $\widehat{H}_{\text{HV}}$ is partitioned into two parts: $\widehat{H}_1(t)$, a single-spin Hamiltonian that depends on time, and $\widehat{H}_2$, the interactions between spins that are time independent.

For the PK algorithm, Wu *et al.* [9] and Yu *et al.* [10] theoretically proposed a seemingly different Hamiltonian,


(4) \begin{eqnarray*}
\widehat{H}_{\text{PK}}(t) = U(t)\widehat{H}_2^{\prime }U^\dagger (t),
\end{eqnarray*}

where $\widehat{H}_2^{\prime }= \sum _{\langle i,j\rangle }V_0\hat{n}_i\hat{n}_j$ and $U(t)=V(t)^{\otimes n}$ with $V(t)$ a ${2\times2}$ unitary matrix. When $V(t)$ changes adiabatically, as specified in [9,10], the MISs are ground states according to the PK algorithm.

## FROM THE SCHRÖDINGER PICTURE TO THE INTERACTION PICTURE

We now demonstrate that Hamiltonians $\widehat{H}_{\text{HV}}$ and $\widehat{H}_{\text{PK}}$ are theoretically equivalent in the sense that $\widehat{H}_{\text{HV}}$ is the Hamiltonian in the Schrödinger picture while $\widehat{H}_{\text{PK}}$ is its counterpart in the interaction picture. The starting point is to note that $\widehat{H}_2^{\prime }$ and $\widehat{H}_2$ are essentially the same for $V_{ij} > 0$, or the repulsive interaction between Rydberg atoms. This is because one can always choose a $V_0 > 0$ such that all $V_{ij} > V_0$. In this case, $\widehat{H}_2^{\prime }$ and $\widehat{H}_2$ have the same set of ground states, which correspond to all independent sets of a given graph. Therefore, only $\widehat{H}_2$ will be referenced in the following discussion.

Consider $\widehat{H}_{\text{HV}} = \widehat{H}_1(t) + \widehat{H}_2$; the evolution of its wave function $\mathinner {|{\Phi _S(t)}\rangle }$ is described by the Schrödinger equation


(5) \begin{eqnarray*}
i\mathrm{d} \mathinner {|{\Phi _S(t)}\rangle }/\mathrm{d} t= [\widehat{H}_1(t)+ \widehat{H}_2]\mathinner {|{\Phi _S(t)}\rangle }.
\end{eqnarray*}

We can move to the interaction picture with the unitary evolution operator $U_I(t) = \mathcal {T}\exp \lbrace -i\int _0^t\hat{{H}}_1(t^{\prime })\mathrm{d}t^{\prime }\rbrace$, and the quantum state $\mathinner {|{\Phi _I(t)}\rangle }$ in the interaction picture is related to the state in the Schrödinger picture as $\mathinner {|{\Phi _I(t)}\rangle }=U_I^\dagger (t)\mathinner {|{\Phi _S(t)}\rangle }$, which satisfies the equation


(6) \begin{eqnarray*}
i\mathrm{d} \mathinner {|{\Phi _I(t)}\rangle }/\mathrm{d} t= U_I^\dagger (t)\widehat{H}_2 U_I(t)\mathinner {|{\Phi _I(t)}\rangle }.
\end{eqnarray*}

When $U_I(t)=U^\dagger (t)$, it is clear that state $\mathinner {|{\Phi _I(t)}\rangle }$ will follow the evolution governed by Hamiltonian $\widehat{H}_{\text{PK}}(t)$, as specified in Equation (4). With the form of $U(t)$ given in [9,10], the condition $U_I(t)=U^\dagger (t)$ allows us to deduce $\widehat{H}_{1}(t)$. If $\widehat{H}_{1}(t)$ takes the same form as $\widehat{H}_{1}(t)$ in Equation (3), Hamiltonian $\widehat{H}_{\text{PK}}(t)$ is equivalent to $\widehat{H}_{\text{HV}}(t)$.

After some calculations, we obtain


(7) \begin{eqnarray*}
\widehat{H}_1 =-i U^\dagger (t)\partial _t U(t)=(\vec{\mu }\times \vec{\mu }^{\prime })\cdot \sum _j\vec{\sigma }_j,
\end{eqnarray*}

where $\vec{\mu }(t)=[\sin (\theta /2) \cos \phi , \sin (\theta /2) \sin \phi , \cos (\theta /2)]$ and $\vec{\mu }^{\prime }(t)=\mathrm{d}\vec{\mu }(t)/\mathrm{d} t$, with $\theta$ and $\phi$ changing with time according to $\theta = \omega _\theta t$ and $\phi = \omega _\phi t\,$. The physical meaning of $\theta$ and $\phi$ can be found in [9,10].

Hamiltonian $\widehat{H}_{1}(t)$ in Equation (7) is of the same form as $\widehat{H}_{1}(t)$ in Equation (3). We have thus shown that Hamiltonian $\widehat{H}_{\text{PK}}(t)$ is equivalent to $\widehat{H}_{\text{HV}}(t)$. In other words, the PK algorithm can be encoded by the Rydberg Hamiltonian with a specific set of $\Omega (t)$, $\varphi (t)$ and $\Delta (t)$.

In the HV experiment [7], the quantum state is in the Schrödinger picture, which as we show above is related to the state in the interaction picture according to $\mathinner {|{\Phi _I(t)}\rangle }=U_I^\dagger (t)\mathinner {|{\Phi _S(t)}\rangle }$. At the end ($t=T$) of the adiabatic evolution specified in [10], we have $\theta =\pi$ and $\phi =0$. This suggests that $U_I^\dagger (T)= U(T) = [\sigma _x ]^{\otimes n}$. Its action is to flip every qubit. For the PK algorithm as discussed in [10], one needs to flip all the spins to get the right answer for the MIS. So the two flips cancel out and we can simply determine the MIS from the Rydberg atom distribution associated with $\mathinner {|{\Phi _S(t)}\rangle }$ finally obtained in the experiment.

## NON-ABELIAN GAUGE MATRIX AND THE PXP MODEL

We note that $-\widehat{H}_1(t)$ is actually a non-Abelian gauge potential. This becomes clear by choosing $U^{\prime }(t)=U(t)\Lambda ^\dagger (t)$ with a unitary $\Lambda (t)$; we find that


(8) \begin{eqnarray*}
-\widehat{H}_1^{\prime } =i \Lambda U^\dagger \partial _t (U\Lambda ^\dagger )= \Lambda (-\widehat{H}_1)\Lambda ^\dagger +i\Lambda \partial _t\Lambda ^\dagger ,
\end{eqnarray*}

which is precisely the gauge transformation of a non-Abelian gauge potential. Furthermore, if we project $-\widehat{H}_1$ onto the subspace of the ground states of $\widehat{H}_2$ (or the independent sets of the corresponding graph), we obtain the non-Abelian gauge matrix *A* of the PK algorithm, namely,


(9) \begin{eqnarray*}
-A(t)=P\widehat{H}_1(t)P,
\end{eqnarray*}

where *P* is the projection onto the ground states of $\widehat{H}_2$. If $V_{ij}\gg \Vert \widehat{H}_1(t) \Vert $, Equation (9) gives an effective Hamiltonian on the subspace of $\widehat{H}_2$ according to the Schrieffer–Wolff transformation. This shows that the Hamiltonian system governed by the gauge matrix *A* is essentially the PXP model [23–29], which is known for its quantum many-body scarring phenomenon and has been experimentally realized with Rydberg atom arrays [30].

The specific correspondence between them is rather straightforward. For the original PXP model in which $\Omega (t)=\Omega$, $\Delta (t)=0$ and $\varphi (t)=0$, the PK algorithm simply sets $\theta (t)=\pi /2$ and $\phi (t)=\Omega t$. We note that quenching the PXP model to find the MIS was proposed recently [33], with an exponentially long runtime due to many-body scars.

The above relation also offers new perspectives on the PXP model, a many-body system originally defined on a one-dimensional chain. We can now view the one-dimensional chain as the underlying graph of the PXP model, with the Néel-type state corresponding to the MIS of this simple graph, and making it possible to extend the PXP model to general graphs. We thus find that, for any graph, there exists a dual graph in which each vertex represents an independent set, and each edge connects a pair of independent sets whose Hamming distance is one (see the appendix for details). This dual graph is a median graph [31,32]. The many-body PXP model, through its relation to *A*, can thus be reinterpreted as a single-particle quantum diffusion over the dual graph. This extension has the potential to enrich the study of quantum many-body scarring. (The oscillating behavior for $\theta =\pi /2$ in Fig. 8 of [9] can be explained accordingly. We will discuss this and related ideas in more detail elsewhere.)

The relationship between *A* and $H_1$ in Equation (9) shows that, when the graph is fixed, the energy gap of *A* is proportional to $-\widehat{H}_1$, which suggests that to have a successful adiabatic path, it is better to let $\widehat{H}_1$ have an as large energy gap as possible. Fortunately, the adiabatic path in the PK algorithm indeed possesses this desirable feature. With $\widehat{H}_1=(\vec{\mu }\times \vec{\mu }^{\prime })\cdot \sum _j\vec{\sigma }_j$, the energy spectrum is simple and the energy gap is $\Delta E = [4\omega _{\phi }^2\sin ^2(\theta /2)+\omega _\theta ^2]^{1/2}$. It is easy to find that, as $\theta$ varies from 0 to $\pi$, $\Delta E$ is steadily increasing and reaches its maximum at the end of the evolution in the PK algorithm, leading to advantages confirmed by the numerical results below.

## NUMERICAL RESULTS

We next compare numerical studies carried out with the two algorithms, mainly addressing two important aspects. One is the comparison of the performance of adiabatic paths, and the other is the analysis of potential accelerations in experiments adopting the PK algorithm versus the HV algorithm. Hamiltonian $\widehat{H}_2$ has many degenerate ground states, associated with the ISs of a graph. The minimum gap between the ISs and the excited states is $V_0$, the interaction strength between Rydberg atoms. When the changing rates $\omega _{\phi }$ and $\omega _{\theta }$ are much smaller than $V_0/\hbar$ in the PK algorithm, the system stays and evolves in the sub-Hilbert space of the degenerate ground states of $\widehat{H}_2$. Its evolution in the sub-Hilbert space is governed by a non-Abelian gauge matrix *A*, which has a minimum energy gap $\delta$. When $\omega _{\theta }/\omega _{\phi }\ll \delta$, at the end of the evolution, $T=\pi /\omega _{\theta }$, the system displays significant amplitudes in states that are either MISs or their good approximations [10]. For the PK algorithm to be effective, adiabatic evolution requires


(10) \begin{eqnarray*}
V_0/\hbar \gg \omega _{\phi }\gg \omega _{\theta }/\delta .
\end{eqnarray*}

**Figure 2.**
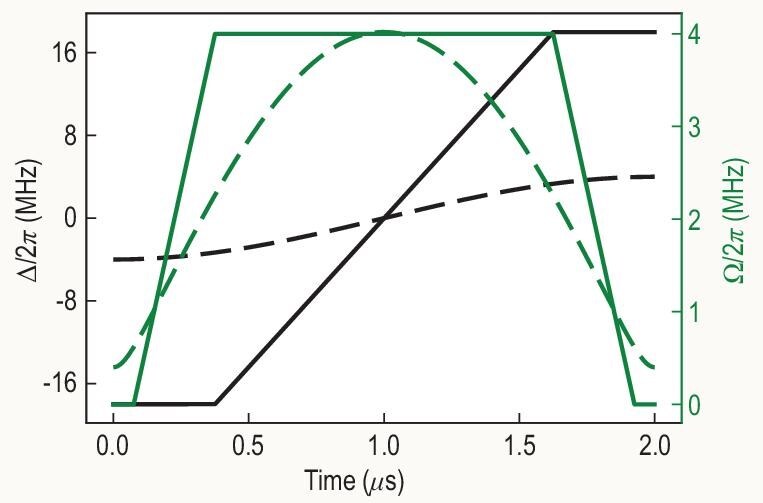
The unoptimized path of the variational quantum adiabatic algorithm is represented by solid lines, the same as the path given in Fig. S8 of [7]. The adiabatic path of the PK algorithm, given by Equations (11a) and (11c), is represented by dashed lines with $\omega _{\theta }=\pi /T$ and $\omega _{\phi }/\omega _{\theta }=-11$.

The explanation and physical origin of the dimensionless energy gap $\delta$ were given earlier in [9,10]. The two changing rates $\omega _{\phi }$ and $\omega _{\theta }$ in the PK algorithm [9,10] are related to the parameters $\Delta$, $\Omega$ and $\varphi$ in the experimental protocol as


(11a) \begin{eqnarray*}
&\Delta (t)=\omega _{\phi } \cos (\omega _{\theta }t),
\end{eqnarray*}

(11b) \begin{eqnarray*}
&\varphi (t)=-\arctan \displaystyle\frac{\omega _{\theta }}{\omega _{\phi }\sin (\omega _{\theta }t)}+\pi ,
\end{eqnarray*}

(11c) \begin{eqnarray*}
&\Omega (t)=\sqrt{\omega _{\phi }^2\sin ^2(\omega _{\theta }t)+\omega _{\theta }^2},
\end{eqnarray*}

which is shown in Fig. 2 as dashed lines. Our analysis below finds that this path exhibits several advantages over those in the HV algorithm discussed in [7].

Two different variational methods are used in [7] to find an optimized evolution path using brute-force classical mathematical search over a restricted set of trial paths. One is called the quantum approximate optimization algorithm (QAOA) [34] and the other is called the variational quantum adiabatic algorithm (VQAA) [35]. For the QAOA, it appears that the experimental protocol (as described above) does not quite implement the optimized evolution path faithfully, which would require an intermittent absence of interaction terms. For the VQAA, it starts with an unoptimized path, shown as solid lines in Fig. 2, and then the path is optimized with the help of a classical computer and experimental inputs.

**Figure 3.**
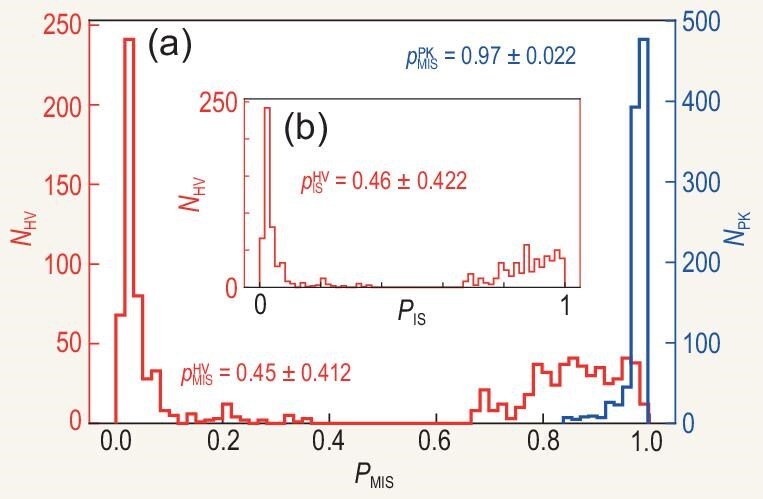
We denote by $P_{\text{MIS}}$ and $P_{\text{IS}}$ the probabilities of finding the MIS and IS, respectively, and by $N_{\text{HV}}$ and $N_{\text{PK}}$ the numbers of graphs employed for running the HV and PK algorithms, respectively. The graphs are 1000 unit disk graphs with seven vertices. The parameters adopted are from the experiment [7], i.e. $V_{\text{NN}}/h=107$ MHz, $V_{\text{NNN}}/h=13$ MHz and $T=1.5$ $\mu$s. (a) The average success rate using the unoptimized path of the HV algorithm is 45% with a standard deviation of 41.2%. Using the adiabatic path of the PK algorithm increases the success rate to 97% with a standard deviation of 2.2%. (b) The average rate of finding independent sets by the HV algorithm is 46% with a standard deviation of 42.2%, which means that in most unsuccessful cases the HV algorithm finds non-independent sets.

The performances of the numerical results are compared for the two paths in Fig. 2 with the seven-vertex graph. On average, we find that the success rate of the PK algorithm is more than twice that of the HV algorithm. With 1000 graphs that are randomly generated, the PK algorithm has an average success rate of 97% and the HV algorithm shows an average success rate of 45%. Further numerical results show that in most cases where the HV algorithm fails, it ends in states of non-independent sets, as shown in Fig. 3(b).

We note that $\varphi (t)=0$ is chosen in our numerical calculation. To implement the PK algorithm faithfully, one actually needs to change $\varphi (t)$ according to Equation (11b). Numerical results show that this does not affect the performance of the PK algorithm. With $\varphi (t)$ following Equation (11b), we find that the PK algorithm reaches an average success rate of 99%.

**Figure 4.**
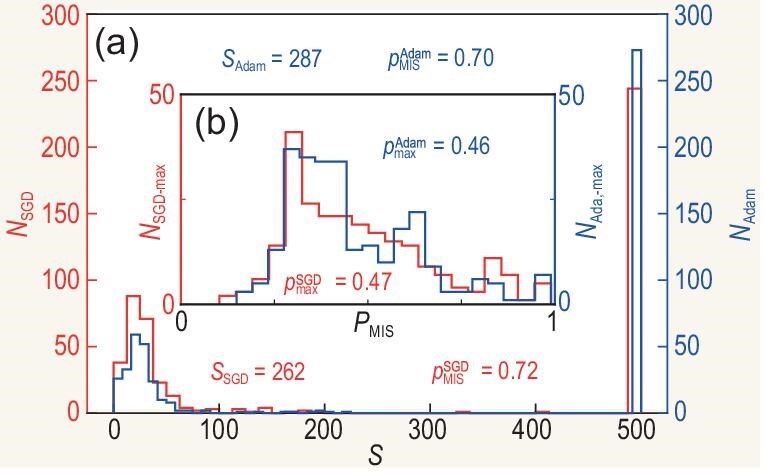
We denote by $S\le 500$ the optimization steps required for the HV algorithm to reach the success rate $\min\, (99\% \ {\rm or}\ P_{\text{MIS}} \text{ of the PK algorithm})$; by $N_{\text{SGD}}$ and $N_{\text{Adam}}$ the numbers of graphs using the SGD and Adam optimizers, respectively; by $P_{\text{MIS}}$ the probability of finding the MIS for the graphs; by $N_{\text{SGD-max}}$ and $N_{\text{Adam-max}}$ the numbers of graphs optimized for 500 steps using the SGD and Adam optimizers, respectively. A total of 500 unit disk graphs with seven vertices are included for each optimizer. (a) The average success rates and average optimization steps are 72% and 262, and 70% and 287, respectively, using the SGD or Adam optimizer. The corresponding percentages of graphs optimized for 500 steps are 48.8% and 54.6%, respectively. (b) For the graphs that reach the maximum optimization steps, the average maximum success rates are 47% with a standard deviation of 19.1% and 46% with a standard deviation of 17.8% using the SGD and Adam optimizers, respectively.

Next, we analyze the potential acceleration of applying the PK algorithm in experiments compared with that of the HV algorithm. The HV algorithm claims that local gradient-based optimizers perform better in the experiment and that Adam’s optimizer performs the best. Thus, we employ the two widely used gradient-based optimizers: stochastic gradient descent (SGD) and Adam. We calculate the number of optimization steps *S* required for the HV algorithm to reach the minimum of 99% or the PK algorithm’s success rate $P_{\text{MIS}}$, with the seven-vertex graph, as shown in Fig. 4.

After 500 steps of optimization using the HV algorithm, employing the SGD or Adam optimizer we find that 48.8% and 54.6% of the graphs, respectively, still fail to reach the optimization target. The average success rates and average optimization steps are 72% and 262, and 70% and 287 for the SGD and Adam optimizers. We conclude that the slightly better performance of the SGD optimizer is due to fine-tuning the learning rate decay during numerical simulations. Assuming that the increase in the success rate is linear, which represents a gross overestimation of the optimization process, it would still take at least 597 steps to reach the performance of the PK algorithm on average. Given that the maximum number of optimization steps in the experiment is about 600 and other limited optimization conditions, the optimized performance of the HV algorithm is significantly outperformed by the PK algorithm in many cases.

Next, we show an experimental significance of the PK algorithm that it can dramatically reduce the number of optimization steps. For a Bernoulli random variable *X* with success probability *p* sampled *m* times, we have the Chernoff bound


(12) \begin{eqnarray*}
\Pr (|\bar{X}-\mathrm{E}(X)|\ge \epsilon )\le 2e^{-2m\epsilon ^2}.
\end{eqnarray*}

For the Rydberg atom experiment, to measure the probability of a certain IS at the end of the evolution, we denote the result differing from the IS as $X=0$ and the result equal to the IS as $X=1$. The number of measurements required to reach a $(1-\eta )$-confidence interval $[p-\epsilon ,p+\epsilon ]$ is then found to be


(13) \begin{eqnarray*}
m\ge \frac{1}{2\epsilon ^2}\log {\frac{2}{\eta }}.
\end{eqnarray*}

For $\epsilon =2\%$, we estimate that this number is at least of the order of $10^3$. Considering that the number of variational parameters in the experiment is ${\sim }10$, every step of the gradient optimization requires at least $10^4$ measurements (${\sim } 1.5$ h of continuous experiment [7]). Given that the PK algorithm only needs one measurement for each graph, it will save about 600 steps of gradient optimization (${\sim }6\times 10^6$ measurements) for one single seven-vertex graph and the algorithm success rate can be improved by at least 25% on average compared to the HV algorithm.

Finally, we analyze the impact of bit-flip errors [36] in the variational process of the HV algorithm, which become negligibly small in the case of the PK algorithm. This reduction arises from the fact that, in the PK algorithm, measurement is performed only on the final state. Assume that a bit-flip error occurs with probability $p_e$ in each measurement. The number of vertices of the graph is *n*, the number of MISs in the graph is denoted by *k*, the probability of the *i*th MIS in the result is denoted by $p_i$ and the probability of all states with Hamming distance *j* away from the *i*th MIS is denoted by $p_{ij}$. With no measurement error, $p_{\text{MIS}0}=p_1+p_2+\cdots +p_k$; in the presence of the above-described measurement error, we have


(14) \begin{eqnarray*}
p_{\text{MIS}}=(1-n p_e)p_{\text{MIS0}} + p_e\sum_{i=1}^{k} p_{i1} + O(p_e^2).
\end{eqnarray*}

The first term in Equation (14) indicates that there will be a decrease in $p_{\text{MIS}}$ proportional to *n*. Assuming that $p_e \sim 10^{-2}$, this result does not have a significant effect on $p_{\text{MIS}}$ when *n* is not very large (${\sim }10^2$). But, in the case of gradient optimization, the effect of the deviation of the gradient will continue to accumulate and amplify as the number of optimization steps increases. This explains why the experimental performance of the HV algorithm oscillates strongly with the number of optimization steps.

## INTRINSIC ADVANTAGES OF THE PK ALGORITHM

From Equations (11a) and (11c), we can see that $\Delta (t)\sim \Omega (t)\sim \omega _{\phi }\ll V_{0}$ in the non-Abelian mixing path. This is an important condition for the evolution to stay in the IS subspace, while in the VQAA, the ansatz $\Delta (t)>V_{0}$, failing to meet this condition. This is corroborated by the average rate of 46% when finding an IS with this ansatz.

Moreover, using the adiabatic theorem, we further find that the adiabatic paths with trigonometric function have intrinsic advantages. With $\Delta (t),\Omega (t)\ll V_{0}$, the Rydberg Hamiltonian can be reduced to a simple effective Hamiltonian


(15) \begin{eqnarray*}
H_{\text{eff}}(t)=-A(t)=\Delta (t)D +\frac{1}{2}\Omega (t)O,
\end{eqnarray*}

where *D* and *O* are constant matrices: $D={\text{diag}}\lbrace n_i\rbrace ={\text{diag}}\lbrace 0,1,1,\dots ,2,2,\dots ,|{\text{MIS}}|\rbrace$ and *O* is an off-diagonal matrix whose nonzero entries are 1 and exist only between states of $\text{HD}=1$. Both *D* and *O* are naturally decided by the IS solutions of the graph. Moreover, *O* is the adjacent matrix of a graph, or the median graph corresponding to the 2-SAT problem (independent set problem), as shown in the appendix.

The empty set is the ground state of $H_{\text{eff}}(0)$ and the MIS is the ground state of $H_{\text{eff}}(T)$; thus, we can consider the adiabatic condition for the ground state of $H_{\text{eff}}(t)$. Since $H_{\text{eff}}(t)$ is real and nonoscillating, the approximate version of the adiabatic condition [37] $\mathinner {\langle {\varepsilon _0(t)|\partial _tH(t)|\varepsilon _1(t)}\rangle }|/{|\varepsilon _1(t)-\varepsilon _0(t)|^2}\ll 1$ is sufficient according to Comparat [38]. Thus, we obtain the following sufficient adiabatic condition for $H_{\text{eff}}(t)$:


(16) \begin{eqnarray*}
&&\frac{|\mathinner {\langle {\varepsilon _0(t)|D|\varepsilon _1(t)}\rangle }|}{\delta ^2(t)}\cdot \bigg |\frac{\Omega \partial _t\Delta -\Delta \partial _t\Omega }{\Omega (\Delta ^2+\Omega ^2)} \bigg |\\
&&\quad\qquad \ll 1 \quad \rm {for\,\,all\,\,t \in [0,T]}
\end{eqnarray*}

with $\delta (t)=[\varepsilon _1(t)-\varepsilon _0(t)]/(\sqrt{\Omega ^2+\Delta ^2})$ the normalized gap. The first term on the left-hand side of Equation (16) denotes the hardness of the graph, which is completely decided by the graph itself. The second term denotes the effect of the experimental parameters, which can be engineered in the experiment, and is preferably small. Taking $\Delta (t)=\omega _{\phi } \cos (\omega _{\theta }t)$ and $\Omega (t)=\omega _{\phi } \sin (\omega _{\theta }t)$, the second term is simplified by $|2\cos (\omega _{\theta }t)\omega _{\theta }/\omega _{\phi }|<2|\omega _{\theta }/\omega _{\phi }|$, which is bounded by a small number due to the intrinsic feature of trigonometric functions.

We thus demonstrate that the PK algorithm, which is a fully quantum algorithm, has a more efficient and resource-saving performance than the HV algorithm, which belongs to a classical-quantum hybrid algorithm. This suggests that the performance of some skillfully constructed adiabatic paths as ours is difficult to match through trivial adiabatic paths combined with brute-force searches.

## MATERIALS AND METHODS

### The median graph for a given graph

**Figure 5.**
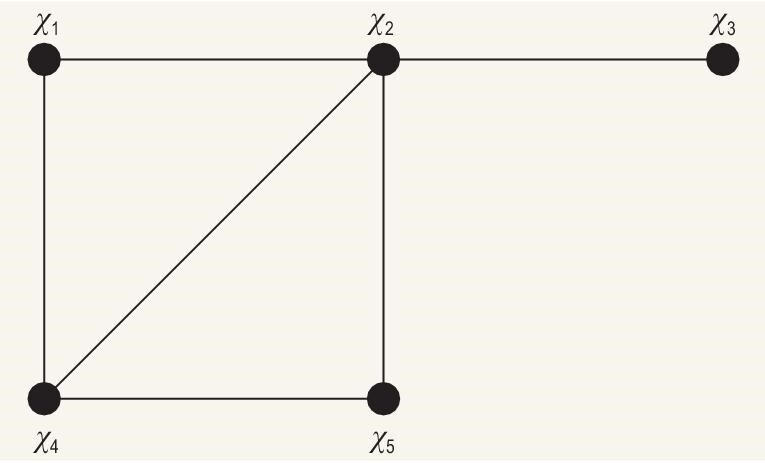
A graph with five vertices and six edges.

For a given graph *G*, there is median graph *g*. The vertices of *g* are all the independent sets of *G*, and the two vertices of *g* are connected if and only if the Hamming distance between the two corresponding independent sets is one. We use the graph in Fig. 5 as an example to illustrate the median graph. We assign each of its vertices a boolean variable. For this example, the five boolean variables are $x_1$, $x_2$, $x_3$, $x_4$ and $x_5$. Each of its independent sets can then be denoted by a binary string. For example, $\lbrace 0,0,0,0,0\rbrace$ represents the empty set, $\lbrace 0,1,0,0,0\rbrace$ represents the independent set with only one vertex $\lbrace x_2\rbrace$, $\lbrace 1,0,1,0,1\rbrace$ represents the maximum independent set $\lbrace x_1,x_3,x_5\rbrace$, etc. As each independent set is denoted by a binary string, we can define the Hamming distance between them as the Hamming distance between the corresponding binary strings. For example, the Hamming distance between the empty set and the set with one vertex $\lbrace x_2\rbrace$ is one; the Hamming distance between the empty set and the MIS $\lbrace x_1,x_3,x_5\rbrace$ is three.

**Figure 6.**
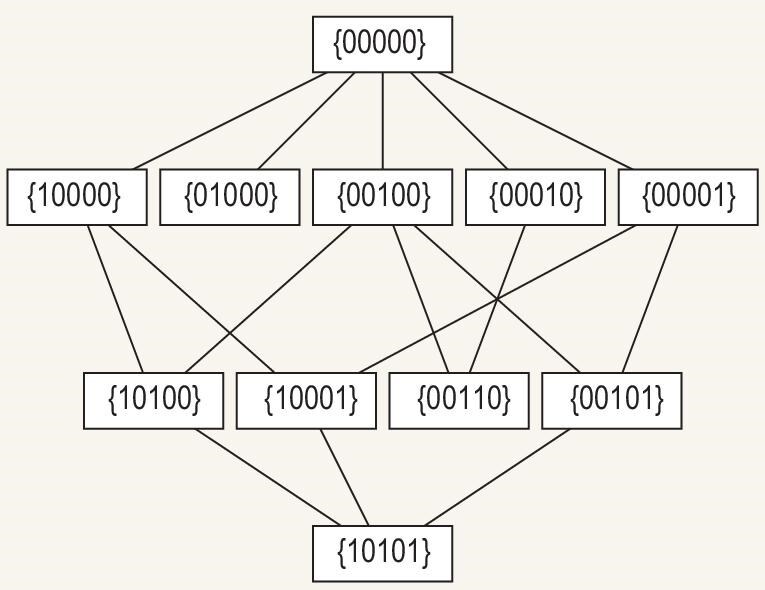
The median graph corresponding to the graph in Fig. 5. Each box (or vertex) represents an independent set.

The graph of Fig. 5 has 11 independent sets, shown in Fig. 6 as marked boxes or vertices of the median graph, that are connected by an edge between a pair of vertices if their Hamming distance is one.
